# Pepper

**Published:** 1857-08

**Authors:** 


### PEPPER

Is an almost universal condiment. Black pepper irritates and inflames the coatings of the stomach, red pepper does not: it excites, but does not irritate, consequently it should be used instead of black pepper. It was known to the Romans, and has been in use in the East Indies from time immemorial, as it corrects that flatulency which attends the large use of vegetable food. Persons in health do not need any pepper in their food. But to those of weak and languid stomachs, it is manifold more beneficial to use Cayenne pepper at meals than any form of wine, brandy, or beer, that can be named, because it stimulates without the reaction of sleepiness or debility.

